# Implementation of a novel nursing assessment tool in geriatric trauma patients with proximal femur fractures

**DOI:** 10.1371/journal.pone.0284320

**Published:** 2023-06-09

**Authors:** Till Berk, Marion Thalmann, Kai Oliver Jensen, Peter Schwarzenberg, Gerrolt Nico Jukema, Hans-Christoph Pape, Sascha Halvachizadeh

**Affiliations:** 1 Department of Trauma, University Hospital Zurich, Zurich, Switzerland; 2 Harald-Tscherne Laboratory for Orthopedic and Trauma Research, University of Zurich, Zurich, Switzerland; 3 AO Research Institute Davos, Davos, Switzerland; University of Verona, ITALY

## Abstract

**Background:**

Geriatric trauma patients represent a special challenge in postoperative care and are prone to specific complications. The goal of this study was to analyse the predictive potential of a novel nursing assessment tool, the outcome-oriented nursing assessment for acute care (ePA-AC), in geriatric trauma patients with proximal femur fractures (PFF).

**Methods:**

A retrospective cohort study of geriatric trauma patients aged ≥ 70 years with PFF was conducted at a level 1 trauma centre. The ePA-AC is a routinely used tool that evaluates pneumonia; confusion, delirium and dementia (CDD); decubitus (Braden Score); the risk of falls; the Fried Frailty index (FFI); and nutrition. Assessment of the novel tool included analysis of its ability to predict complications including delirium, pneumonia and decubitus.

**Results:**

The novel ePA-AC tool was investigated in 71 geriatric trauma patients. In total, 49 patients (67.7%) developed at least one complication. The most common complication was delirium (n = 22, 44.9%). The group with complications (Group C) had a significantly higher FFI compared with the group without complications (Group NC) (1.7 ± 0.5 vs 1.2 ± 0.4, p = 0.002). Group C had a significantly higher risk score for malnutrition compared with Group NC (6.3 ± 3.4 vs 3.9 ± 2.8, p = 0.004). A higher FFI score increased the risk of developing complications (odds ratio [OR] 9.8, 95% confidence interval [CI] 2.0 to 47.7, p = 0.005). A higher CDD score increased the risk of developing delirium (OR 9.3, 95% CI 2.9 to 29.4, p < 0.001).

**Conclusion:**

The FFI, CDD, and nutritional assessment tools are associated with the development of complications in geriatric trauma patients with PFF. These tools can support the identification of geriatric patients at risk and might guide individualised treatment strategies and preventive measures.

## Introduction

Adults over the age of 65 years account for the fastest growing population segment. In the United States, this population group will double to 89 million between 2010 and 2050 [1]. This is reflected in the current development of patient demographics where geriatric patients represent a special challenge for medical treatment: they often present with polypharmacy, comorbidities and increased risk of complications [2]. Furthermore, the increasing number of the elderly population has led to a significant increase in the incidence of geriatric patients with fractures [3]. Proximal femur fractures (PFF) are among the most common injuries in the geriatric population; they require hospitalisation and surgical treatment. In 1990, the global number of reported hip fractures was 1.3 million, and this number is expected to increase to between 7.3 and 21.3 million by 2050 [3]. Geriatric patients with fractures are at increased risk of morbidity and mortality and have an increased need for intensive care or permanent institutionalisation [4, 5].

Postoperative complications influence the course and outcome following surgery and are associated with increased socioeconomic burden [6–8]. A suitable risk assessment score, targeting prophylactic measures and early detection of warning signs, could reduce complications and costs [7]. The outcome-oriented nursing assessment for acute care (ePA-AC) aims to predict special nursing requirements and assistance. It is used in approximately 500 medical institutions in Germany, Switzerland and Austria, and specific versions are available for paediatrics, long-term care and psychiatry wards [9, 10]. Currently, over 210,000 people in almost 800 institutions across Europe are using this assessment tool [11]. However, there are only a few independent clinical studies on the validity and practicability of the ePA-AC [9, 12, 13], and these studies have been generalised to heterogeneous patient collectives and a wide range of hospitalisation indications.

The aim of this study was to analyse the implementation of the novel ePA-AC nursing tool in geriatric trauma patients with PFF who had undergone surgical treatment.

## Methods

### Ethical considerations

The protocol of this study was approved by the institutional review board "Cantonal Ethics Committee Zurich" (No. 2019–01957). Since 2016, all patients have been required to sign an informed consent form during their hospitalisation that allows the anonymous utilisation of any collected data in future studies. Therefore, written informed consent was obtained from all participants for inclusion in the study. Consequently, the present study only included data from patients who signed and approved the utilisation of the anonymised medical data during their initial hospitalisation. The data were anonymised and extracted from the electronic medical record for this retrospective cohort study.

### Study population

This retrospective cohort study included geriatric trauma patients who had had surgical treatment of a PFF at a single academic level 1 trauma centre in 2017. Patients with genetic disorders affecting the musculoskeletal system, as well as patients with oncologic diseases, were excluded from this study. Furthermore, patients with additional ipsilateral upper limb injuries, open injuries, associated vascular injuries as well as nerve damage and patients requiring more than one surgery were excluded. Additional exclusion criteria included the presence of multiple injuries, patients who had received surgery from a foreign hospital before being transferred and pre-existing decubiti.

Patients were stratified according to the development of complications during their hospital stay into a group with complications (Group C) and a group without complications (Group NC).

### The ePA-AC score

The ePA-AC was developed in Wiesbaden, Germany, in 2002 as part of a research project and has been continuously improved since its creation. The aim was to determine several key figures for nursing needs and specific risk factors from routine nursing assessments to optimise nursing care planning and to more easily identify patients at risk of certain complications. Furthermore, the repeated collection of data over the course of the inpatient stay allows an assessment of the patient’s progress. The ePA-AC performed on the day of admission and the day of discharge by the nursing staff on the ward was used for this study. Therefore we included the first and the last documented variables. The ePA-AC consists of over 50 items in 10 categories: Movement, Personal Care & Dress, Nutrition, Elimination, Cognition/Consciousness, Communication/Interaction, Sleep, Respiration, Pain, and Pressure Ulcers. Each item is assigned either one-of-two or one-of-four proficiency levels according to criteria clearly defined in the manual. The associated software converts these into numerical values and calculates, among other things, the ‘Self Care Index’ (SCI) [14], the ‘Braden Score’ (BS) [15], the ‘risk of pneumonia’, the ‘risk of development of confusion/delirium/dementia’ (CDD) [16], the ‘Mini Nutritional Assessment’ (MNA) [17], the ‘risk of malnutrition’ (MN) [18] and the ‘risk of falls’, based on stored cut-off values (S1 and S2 Files).

### Definitions and assessment tools

Complications included urinary tract infection, pneumonia, deep surgery site infection, decubitus, anaemia that required blood transfusions and delirium that were recorded during hospitalisation (Table 1).

**Table 1:** Complications.

**Urinary tract infection, n (%)**	12 (16.9)
**Pneumonia, n (%)**	7 (9.9)
**Blood transfusion, n (%)**	21 (29.6)
**Delirium, n (%)**	22 (31.0)
**Decubitus, n (%)**	10 (14.1)
**Highest Clavien-Dindo grade, n (%)**	
**0**	5 (7.0)
**1**	11 (15.5)
**2**	45 (63.4)
**3a**	1 (1.4)
**3b**	3 (4.2)
**4a**	3 (4.2)
**5**	3 (4.2)
**CoCoI, mean (SD)**	30.88 (20.86)

CoCoI = Comprehensive Complication Index; n = number; SD = standard deviation

The Clavien-Dindo (CD) Classification was utilised as described previously [19]. In brief, the classification system stratifies complications according to the invasiveness of the treatment as grade 1 (any deviation from the normal postoperative course with observational treatment), grade 2 (requiring pharmacological treatment of the complication), grade 3 (requiring interventional or surgical treatment), grade 4 (life-threatening complication) and grade 5 (death). The Charlson Comorbidity Index (CCI) was used to quantify comorbidities [20]. PFF included fractures of the femoral neck as well as intertrochanteric and subtrochanteric fractures.

The in-hospital assessment of frailty was performed with Fried Frailty Index (FFI) and malnutrition was assessed with the Mini Nutritional Assessment Score (MNA); these values were obtained from the geriatric assessment [17, 21, 22]. The ePA-AC was assessed on admission by the nursing staff and repeated daily over the course of the inpatient stay.

### Treatment protocol

All geriatric trauma patients were treated according to our in-house protocol following a collaboration of geriatricians and trauma surgeons as described previously [23]. The surgical treatment included nail osteosynthesis, dynamic hip screws/screw osteosynthesis or a total joint replacement. Postoperatively, patients had regular physiotherapeutic mobilisation that began, at the latest, on the day after surgery.

### Statistical analysis

Continuous variables are summarised as mean with standard deviation (± SD) while categorical variables are presented as counts and percentages. Student’s t-test was used to compare two groups of continuous variables, while the chi-squared test was used to compare groups of binary variables. Generalised mixed model regression analyses were performed to calculate the odds ratio (OR) for the development of complications (binomial outcome). Variables for multivariable analyses were chosen based on exploratory analyses (p ≤ 0.1) and variables that are relevant based on clinical experience. In addition, the assessment tools were analysed for their sensitivity and specificity. Therefore, the predicted and observed variables were compared. R (Version 3.3.6, R Foundation for Statistical Computing, Vienna, Austria) was used for all calculations, analyses and the preparation of the graphical representations. Statistical significance was set at an alpha value of < 0.05.

## Results

This study included 71 patients with a mean age of 83.5 (± 7.8) years, and 24 (33.8%) patients were male. The majority of patients (n = 67, 94.4%) suffered a low-energy ground-level fall and the mean CCI was 5.1 (± 1.6) points. Overall, 48 (67.6%) patients experienced at least one complication (Table 2). The most common complication was postoperative delirium (n = 22, 31.0%), followed by the requirement of blood transfusion (n = 21, 29.6%) and urinary tract infections (n = 12, 16.9%). Most patients had a CD 2 complication (n = 45, 63.4%), followed by a CD 1 complication (n = 11, 15.5%). The mean Comprehensive Complication Index (CoCoI) was 30.9 ± 20.9 points.

**Table 2:** Patients demographics.

**n**	71
**Age [years], mean (SD)**	83.54 (7.78)
**Male sex, n (%)**	24 (33.8)
**Injury, n (%)**	
**Femoral neck fracture**	33 (46.5)
**Femur fracture, inter and sub-trochanteric**	38 (53.5)
**CCI [points], mean (SD)**	5.06 (1.64)
**LOS [days], mean (SD)**	14.85 (7.62)
**Delirium, n (%)**	20 (28.2)
**Trauma mechanism, n (%)**	
**Low energy**	67 (94.4)
**Moderate energy**	2 (2.8)
**High energy**	2 (2.8)
**In-hospital death, n (%)**	3 (4.2)
**Complications, n (%)**	48 (67.6)

CCI = Charlson Comorbidity Index; LOS = length of stay; n = number; SD = standard deviation.

Groups C and NC were comparable in age, sex distribution and the CCI, with no significant differences between the two groups. The MNA was significantly lower in Group C compared with Group NC (18.6 ± 5.3 vs 22.1 ± 3.3 p = 0.037) and the risk of malnutrition was significantly higher in Group C (6.3 ± 3.4 vs 3.9 ± 2.8 points, p = 0.001). Furthermore, the FFI was significantly higher in Group C (1.7 ± 0.5) compared with Group NC (1.2 ± 0.4, p = 0.002). Moreover, Group C had a significantly higher CDD than Group NC (22 ± 45.8 vs 3 ±13.0 p = 0.015) (Table 3).

**Table 3:** Baseline evaluation of geriatric patients stratified according to the development of complications.

	Group NC	Group C	p
	23	48	
**Age [years], mean (SD)**	81.61 (8.04)	84.46 (7.57)	0.15
**Male sex, n (%)**	9 (39.1)	15 (31.2)	0.697
**BMI [kg/m** ^**2**^ **], mean (SD)**	23.13 (3.43)	23.71 (4.42)	0.594
**CCI [points], mean (SD)**	4.65 (1.56)	5.25 (1.66)	0.151
**LOS [days], mean (SD)**	11.52 (6.66)	16.44 (7.59)	0.01
**CoCoI [points], mean (SD)**	13.27 (9.01)	39.31 (19.64)	<0.001
**Fried Frailty Index [points], mean (SD)**	1.21 (0.43)	1.73 (0.46)	0.002
**ePA-AC assessment**			
**SCI [points], mean (SD)**	26.57 (5.32)	26.44 (12.04)	0.961
**Braden Score [points], mean (SD)**	18.48 (17.69)	16.54 (12.37)	0.595
**Risk of pneumonia, n (%)**	23 (100.0)	45 (93.8)	0.552
**CDD, n (%)**	3 (13.0)	22 (45.8)	0.015
**MNA [points], mean (SD)**	22.12 (3.28)	18.62 (5.32)	0.037
**Risk of malnutrition [points], mean (SD)**	3.87 (2.78)	6.31 (3.40)	0.004

BMI = body mass index; Braden Score = decubitus risk; CCI = Charlson Comorbidity Index; CDD = risk of confusion, delirium and dementia; CoCoI = Comprehensive Complication Index; ePA-AC = outcome-based nursing assessment for acute care; LOS = length of stay; MNA = Mini Nutritional Assessment; SCI = self-care index.

A higher FFI score increased the risk of developing complications (OR 9.8, 95% confidence interval [CI] 2.00 to 47.7, p < 0.005). A higher MNA score lowered the risk of developing complications (OR 1.18, 95% CI 0.802 to 1.726) (Table 4).

**Table 4:** Predicting complications.

		95% confidence interval		
	**OR**	**Lower**	**Upper**	**p**
**Fried Frailty Index [points], mean (SD)**	9.78	2.00	47.7	0.005
**MNA [points], mean (SD)**	1.18	0.802	1.726	0.405
**SCI [points], mean (SD)**	1.0	0.693	1.572	0.836
**Braden Score [points], mean (SD)**	0.89	0.365	2.204	0.813
**CDD**	2.328	0.881	6.151	0.088
**Risk of malnutrition [points], mean (SD)**	0.929	0.616	1.401	0.725

Braden Score = decubitus risk; CDD = risk of confusion, delirium, dementia; Braden = decubitus risk; CDD = risk of confusion, delirium, dementia; MNA = Mini Nutritional Assessment; SCI = Self-care Index; MNA = Mini Nutritional Assessment; OR = odds ratio; SCI = Self-care Index.

The sensitivity of the ‘Risk of pneumonia’ assessment tool was 0.13 (95% CI 0.05 to 0.24) (Table 5). A higher BS increased the odds of developing decubitus by 6.2 times (95% CI 1.5 to 25.7, p < 0.001). Patients who were at risk of CDD had 9.3 times increased odds of developing delirium (95% CI 2.9 to 29.4, p < 0.001).

**Table 5:** Epidemiological analysis of risk factors assessed by the outcome-based nursing assessment for acute care (ePA-AC).

	Risk of pneumonia	95% confidence interval	Risk of delirium	95% confidence interval
**Sensitivity**	0.13	0.31 to 0.47	0.41	0.28 to 0.55
**Specificity**	0.26	0.18 to 0.37	0.44	0.34 to 0.55
**Positive predictive value**	0.10	0.04 to 0.19	0.31	0.21 to 0.43
**Negative predictive value**	0.32	0.22 to 0.45	1.34	0.97 to 1.85
**Positive likelihood ratio**	0.7	0.09 to 0.35	0.73	0.50 to 1.06
**Negative likelihood ratio**	3.3	2.29 to 4.75	1.73	0.97 to 0.85

## Discussion

The increasing number of geriatric patients with fractures represents a special challenge in orthopaedic trauma care. Risk assessment tools have been developed to identify geriatric patients at risk of complications. This study aimed to assess the predictive value of the ePA-AC on the development of complications in geriatric trauma patients and found the following:

1. Two out of three geriatric patients developed at least one complication, the most common being delirium (31%).
2. The FFI and malnutrition were associated with the development of complications.
3. The BS and CDD were associated with the development of decubitus and delirium.

Studies on geriatric trauma patients have reported complication rates of 28.4%-39.5% [24, 25]. However, the present study population is older (83.54 ± 7.78 years) compared with the previously mentioned studies (77.9 and 78.2 years) [24, 25]. Additionally, a large Dutch study from 2018 with 479 included patients found a complication rate of 75% in the first 6 months after surgery with a mean age of 78.4 years of the study population [26].

The increased rate of delirium might reflect a common perioperative complication. The prevalence of delirium has been reported to range from 30% in older hospitalised patients, up to 92% in mechanically ventilated trauma patients [27–29]. Early detection and appropriate treatment of elderly patients at risk of delirium can improve care of this population [30]. This study showed that CDD obtained from the ePA-AC was associated with the development of complications in this sector and might identify patients at risk of delirium and guide appropriate management strategies [31].

The MNA and the FFI are associated with the development of in-hospital complications. Geriatric patients might suffer from a progressive reduction of muscle mass that might impair postoperative outcome [32–34]. The MNA focuses on the nutritional status and might represent a more holistic assessment. Its high sensitivity has already been recognised in the literature [35]. The FFI is associated with an increased risk of complications. This result is in accordance with previous studies on geriatric trauma patients and their complication predictions with the FFI [24, 36–40]. In the literature, the FFI has also been shown to be predictive of the development of pneumonia [41, 42]. Therefore, the targeted, combined use of multiple scores, aimed to the patient population under investigation, may provide the best predictive power.

The number of frail patients has been reported to range from 10.7% to 15.7% (the 80-84-year-old group) and 26.1% (> 85-year-old group) [43]. The discrepancies in these numbers suggest a lack of consensus in the definition of frailty [44] based on the enormous variety of frailty assessment tools, with variability in terms of scoring and evaluated domains [45, 46]. However, the prevalence of frailty is reported to be higher in studies that used multidimensional assessment tools [44]. The results of this study have shown that the ePA-AC could represent such a multidimensional assessment tool–especially because it seems that the search for an ideal score for the assessment of elderly patients has not yet been achieved. Similarly, the definition and screening of delirium represent a challenge in current medical treatment, leading to the utilisation of different tools in different situations: CAM assessment in intensive care unit patients, or MDAS in cancer patients [47]. CDD might be a promising tool for detecting geriatric trauma patients at risk of delirium.

### Strengths and limitations

The retrospective design provides certain well-known limitations. The nature of the study design aims to include the maximum available dataset. Therefore, a formal sample size calculation was not performed. However, a power analysis might indicate the risk for a potential type 2 error. A certain effect size would be required to calculate both the sample size and the power of the present study. Because the present study has investigated a novel tool, the effect size would have to be drawn from the present data and the results of both the sample size calculation and the power would be biased and challenging to interpret. However, we believe that based on the standardised treatment protocol and the comparability of the study groups, the presented results provide some evidence for the association between the assessment tools and the development of complications. With many years of practical application and a large number of users and institutes, the ePA-AC seems to be a well-established tool for patient assessment with recognisable potential to predict complications in geriatric trauma patients.

One might argue that the severity of injury or the surgical intervention might present a risk factor for the development of complications. This study included only proximal femur fractures to minimize the effect of the injury-variability or the effect of surgery on our outcome parameter.

Another limitation is the inability to evaluate the scores for pneumonia, especially because pneumonia is a frequent complication in this patient population. Given that the data were collected from a certified centre for geriatric traumatology, with well-established treatment pathways, it can be assumed that all patients were treated according to the same medical standards and with the best possible care given. Furthermore, patients with polymorbidity in poor general condition are often referred to a level 1 centre for geriatric traumatology such as ours. These factors could explain the high number of complications with a potential selection bias. Lastly, the size of the cohort is considered small. Nevertheless, the comparability of geriatric patients is challenging due to pre-existing conditions, multiple injuries and previous surgeries. To achieve the best possible comparability of the included patients, only mono-traumatised patients with comparable fractures were included. The price for this comparability was a small cohort. Additional studies with larger cohorts should be conducted in order to be able to evaluate the findings from this study.

## Conclusion

The FFI has the highest predictive value for an increased risk of developing complications in general. CDD is a promising tool for identifying geriatric trauma patients at risk of delirium. Utilisation of the appropriate assessment tool for geriatric trauma patients might support individualised treatment strategies.

## Supporting information

S1 File Outcome-based nursing assessment for acute care (ePA-AC): Items by category with relevance for risk assessments. (PPTX)

S2 File Outcome-based nursing assessment for acute care (ePA-AC): Items by category with relevance for risk assessments. (PPTX)

S1 Dataset (CSV)
