# Supplementary material for: Implementation of a novel nursing assessment tool in geriatric trauma patients with proximal femur fractures
Source: PLoS One. 2023 Jun 9;18(6):e0284320. doi: 10.1371/journal.pone.0284320 (PMC10256203; doi:10.1371/journal.pone.0284320)
Supplement: S1 File — (PPTX) [file pone.0284320.s001.pptx]

## Slide 1
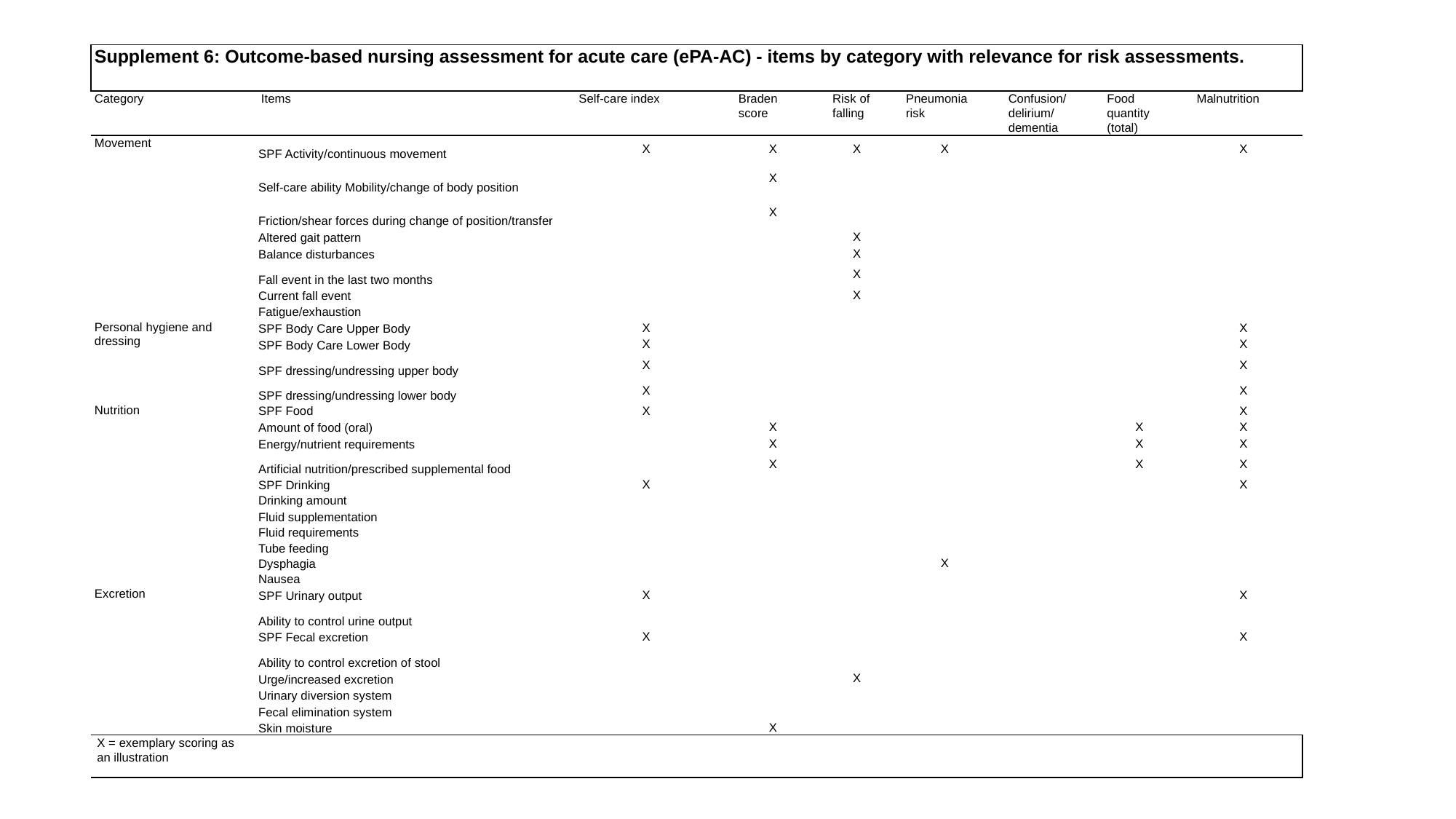

| Supplement 6: Outcome-based nursing assessment for acute care (ePA-AC) - items by category with relevance for risk assessments. | | | | | | | | |
| --- | --- | --- | --- | --- | --- | --- | --- | --- |
| Category | Items | Self-care index | Braden score | Risk of falling | Pneumonia risk | Confusion/ delirium/ dementia | Food quantity (total) | Malnutrition |
| Movement | SPF Activity/continuous movement | X | X | X | X | | | X |
| | Self-care ability Mobility/change of body position | | X | | | | | |
| | Friction/shear forces during change of position/transfer | | X | | | | | |
| | Altered gait pattern | | | X | | | | |
| | Balance disturbances | | | X | | | | |
| | Fall event in the last two months | | | X | | | | |
| | Current fall event | | | X | | | | |
| | Fatigue/exhaustion | | | | | | | |
| Personal hygiene and dressing | SPF Body Care Upper Body | X | | | | | | X |
| | SPF Body Care Lower Body | X | | | | | | X |
| | SPF dressing/undressing upper body | X | | | | | | X |
| | SPF dressing/undressing lower body | X | | | | | | X |
| Nutrition | SPF Food | X | | | | | | X |
| | Amount of food (oral) | | X | | | | X | X |
| | Energy/nutrient requirements | | X | | | | X | X |
| | Artificial nutrition/prescribed supplemental food | | X | | | | X | X |
| | SPF Drinking | X | | | | | | X |
| | Drinking amount | | | | | | | |
| | Fluid supplementation | | | | | | | |
| | Fluid requirements | | | | | | | |
| | Tube feeding | | | | | | | |
| | Dysphagia | | | | X | | | |
| | Nausea | | | | | | | |
| Excretion | SPF Urinary output | X | | | | | | X |
| | Ability to control urine output | | | | | | | |
| | SPF Fecal excretion | X | | | | | | X |
| | Ability to control excretion of stool | | | | | | | |
| | Urge/increased excretion | | | X | | | | |
| | Urinary diversion system | | | | | | | |
| | Fecal elimination system | | | | | | | |
| | Skin moisture | | X | | | | | |
| X = exemplary scoring as an illustration | | | | | | | | |
