# Supplementary figures and images for: Implementation of a novel nursing assessment tool in geriatric trauma patients with proximal femur fractures
Source: PLoS One. 2023 Jun 9;18(6):e0284320. doi: 10.1371/journal.pone.0284320 (PMC10256203; doi:10.1371/journal.pone.0284320)

## Slide 1
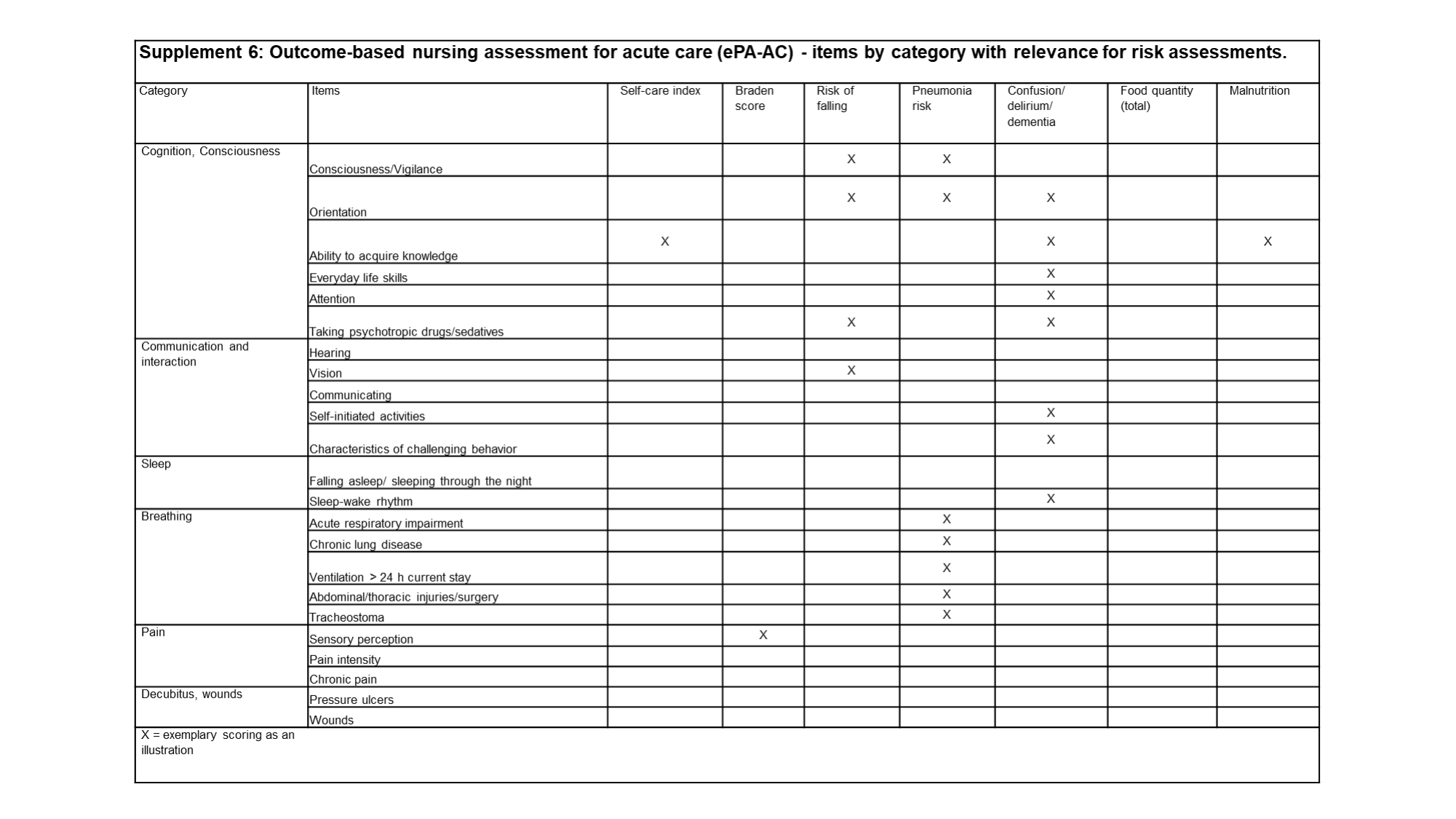

Supplement: S2 File — (PPTX) [file pone.0284320.s002.pptx]
